# A combinational CRISPR/Cas9 gene-editing approach can halt HIV replication and prevent viral escape

**DOI:** 10.1038/srep41968

**Published:** 2017-02-08

**Authors:** Robert Jan Lebbink, Dorien C. M. de Jong, Femke Wolters, Elisabeth M. Kruse, Petra M. van Ham, Emmanuel J. H. J. Wiertz, Monique Nijhuis

**Affiliations:** 1 Department of Medical Microbiology, Virology, University Medical Center Utrecht, Utrecht, The Netherlands

## Abstract

HIV presents one of the highest evolutionary rates ever detected and combination antiretroviral therapy is needed to overcome the plasticity of the virus population and control viral replication. Conventional treatments lack the ability to clear the latent reservoir, which remains the major obstacle towards a cure. Novel strategies, such as CRISPR/Cas9 gRNA-based genome-editing, can permanently disrupt the HIV genome. However, HIV genome-editing may accelerate viral escape, questioning the feasibility of the approach. Here, we demonstrate that CRISPR/Cas9 targeting of single HIV loci, only partially inhibits HIV replication and facilitates rapid viral escape at the target site. A combinatorial approach of two strong gRNAs targeting different regions of the HIV genome can completely abrogate viral replication and prevent viral escape. Our data shows that the accelerating effect of gene-editing on viral escape can be overcome and as such gene-editing may provide a future alternative for control of HIV-infection.

HIV, like other RNA-viruses, presents one of the highest mutation rates observed in nature^1^,^2^. In addition to this, HIV has a fast replication rate and generates a large population size, all accelerating viral evolution. As such, the viral population within an infected individual consists of a swarm of mutant viruses also referred to as a quasispecies. The challenge in the treatment of HIV infection is to overcome the plasticity of the virus. The current therapeutic arsenal consists of more than 25 different antiretroviral compounds that inhibit different steps in the HIV life cycle. When used in combination, these antiretroviral drugs can profoundly control viral replication by preventing pre-existing mutants from replicating and acquiring additional resistance mutations^3^,^4^,^5^. However, treatment intensification studies and viral reservoir analysis indicate that low-level viral replication may persist in some individuals even after long-term cART^6^,^7^,^8^. Furthermore, current antiretroviral compounds don’t target the integrated provirus nor suppress HIV expression and production from the cellular reservoir. Continuous viral replication and/or viral production contribute to persistent inflammation, activation and immune dysfunction, causing a wide-range of morbidities in the aging patient population^9^,^10^. HIV-induced inflammation and activation will in turn contribute to viral production and proliferation of infected cells, reinforcing this vicious cycle. As a result, HIV will persist indefinitely in the infected individual and needs to be suppressed with lifelong therapy.

Alternative strategies are urgently needed to completely stop viral production and replication in the viral reservoir. By directly disrupting the proviral genome within this reservoir, the virus may be eliminated from the host and a cure would be achieved. Over the last decade, several nucleases have been successfully engineered for genome modifications, such as zinc finger nucleases (ZFNs)^11^, transcription activator-like effector nucleases (TALENs)^12^ and more recently the clustered regularly interspaced palindromic repeat (CRISPR) Cas9 nuclease^13^,^14^,^15^. These nucleases can induce double-strand DNA breaks at defined target sites, which are subsequently repaired by the error-prone non-homologous end joining (NHEJ) machinery. Upon repair of the target site, a high incidence of insertions and deletions (indels) and nucleotide substitutions around the target site are yielded^16^.

ZFNs already showed promising, albeit differential results, in a proof of concept clinical trial where autologous CD4^+^T cells with ZFN-induced mutations in the CCR5 co-receptor were infused in HIV infected individuals^17^. Conversely, as ZFNs and TALENs exhibit a relatively low targeting efficiency, are difficult to design and exert some ambiguity in their activity^18^,^19^, their use remains challenging. The recently developed CRISPR/Cas9 system, however, has gained much momentum and is now commonly considered as a superior strategy for directed genome engineering. CRISPR/Cas9 gRNAs are relatively quick and easy to design and co-expression with the Cas9 endonuclease has previously proven effective in targeting double stranded DNA viruses^20^,^21^,^22^,^23^,^24^,^25^ and viruses with a double stranded DNA intermediate such as HBV^26^,^27^,^28^,^29^,^30^,^31^,^32^,^33^,^34^ and HIV^35^,^36^,^37^,^38^,^39^,^40^,^41^.

Unlike most dsDNA viruses, HIV exhibits extensive antiviral drug escape. Here, we investigate whether and how escape from CRISPR/Cas9 targeting of single or multiple steps in the viral life cycle may occur. In line with two recent studies^42^,^43^, we demonstrate that targeting of the HIV provirus at a single locus only partially inhibits HIV replication and facilitates rapid viral escape by selection of sequence variants at the target site. Here we report that a combinatorial CRISPR/Cas9 gene-editing approach where two different regions of the viral genome are simultaneously targeted, can halt HIV replication and prevent viral escape. These findings indicate that HIV escape can be overcome and as such gene-editing may provide a future alternative for control of infection.

## Results

### Efficient targeting and editing of HIV by CRISPR/Cas9

**Figure 1:**
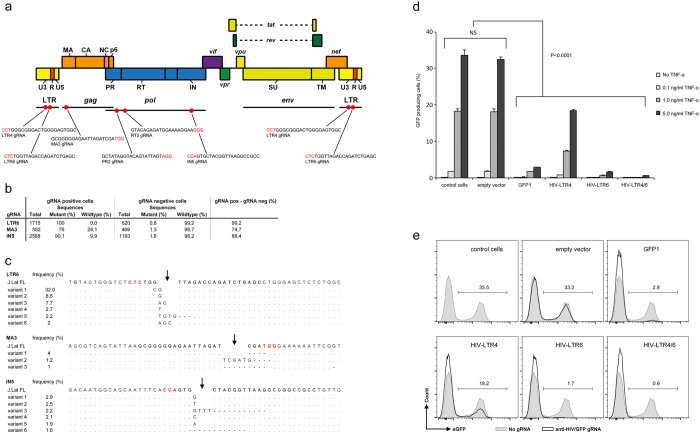
Efficient CRISPR/Cas9 targeting of HIV-LTR abrogates HIV gene expression. (**a**) Schematic representation of the HIV genome and the location of the anti-HIV gRNA target sequences. Target sequences were designed based on a reference HIV-1 genome (HXB2) and all sequences initiated with a G-residue to allow for potent expression from the U6 promoter. We allowed for the presence of both NGG and NAG PAM sequences (indicated in red) adjacent the target site. (**b,c**) Sequencing of the HIV target region indicates efficient editing of HIV DNA in latently infected cells. Total number of reads, and percentage mutant and wildtype sequence in the target region of the CRISPR/Cas9 endonuclease (three nucleotides upstream and downstream of the cleavage site) is shown in gRNA positive cells and gRNA negative control cells (**b**). The most frequently observed HIV mutants from B) are shown (**c**). We presented the most dominantly observed variants with a frequency above 1% of the total number of reads. The Cas9 cleavage site is indicated with an arrow and the PAM sequence is presented in red. (**d,e**) Efficient targeting of HIV-LTR in latently infected cells abrogates HIV gene expression. The J.Lat FL cells were stably transduced with lentiviral vectors targeting the HIVLTR region. Upon induction with TNF-α, reactivation of HIV was monitored by analysis of GFP reporter-expression. Data is presented as a mean with standard deviation from three or more independent assays (**d**). One representative experiment is indicated (**e**).

We first assessed the ability of stably expressed gRNAs to target and edit HIV DNA. Two gRNAs sequences designed to target the HIV-1 LTR region were co-expressed in a lentiviral vector with the Cas9 endonuclease (Fig. 1a and Supplementary Table S1). Additionally, gRNAs were designed to target the structural viral matrix protein, protease, reverse transcriptase (RT), and integrase, all essential for viral replication (Fig. 1a, Supplementary Table S1). We infected Jurkat cells containing a near full length copy of HIV (J.Lat FL cells; J.Lat Full Length Clone 15.4; HIV-R7/E-/GFP) with the lentiviruses containing a gRNA targeting the LTR region (LTR6), the structural protein matrix (MA3) or the integrase enzyme (IN5). Deep sequence analysis showed specific genome editing events at the corresponding target site in 100%, 76% and 90.1% of the sequences for respectively the gRNAs LTR6, MA3 and IN5 (Fig. 1b; Supplementary Table S2). The most frequently observed variants contained insertions or deletions at the target site as compared to the wildtype sequence (Fig. 1c). As a control we also sequenced the target regions in absence of CRISPRs (gRNA negative J.Lat FL cells) and observed between 0.8–1.8% sequence changes in this region, which were likely introduced by amplification or sequencing errors because comparable (number of) sequence changes were also observed if we analyze a random sequence nearby the gRNA target sequence (Fig. 1b, Supplementary Table S2).

### Efficient CRISPR/Cas9 targeting of HIV-LTR abrogates HIV gene expression

Next, we investigated whether disruption of the viral genome sequence by the CRISPR/Cas9 system would also abrogate gene expression. In the above-described model, the integrated HIV-genome is complemented with an HIV LTR driven GFP gene which becomes expressed upon TNF-α the activation. We therefore focused on the LTR region and selected two gRNAs, LTR4 and LTR6, that target the SPI binding region and TAR loop, respectively (Fig. 1a, Supplementary Table S1). Their individual and combined ability to abrogate HIV (GFP) expression was investigated. Upon induction with TNF-α, we observed reactivation in up to 35% of the latently infected cells (control J.Lat FL cells and control cells transduced with an empty vector) (Fig. 1d,e). Upon introduction of anti-LTR gRNAs, reactivation from latency was significantly reduced (p < 0.0001), as gRNA LTR4 resulted in a 40% loss and gRNA LTR6 in a 95% loss of GFP reporter-expression. Combining these two anti-LTR gRNAs enhanced the loss of eGFP expression to > 98% (Fig. 1d,e).

### Single anti-HIV gRNAs cannot circumvent HIV replication and viral escape

**Figure 2:**
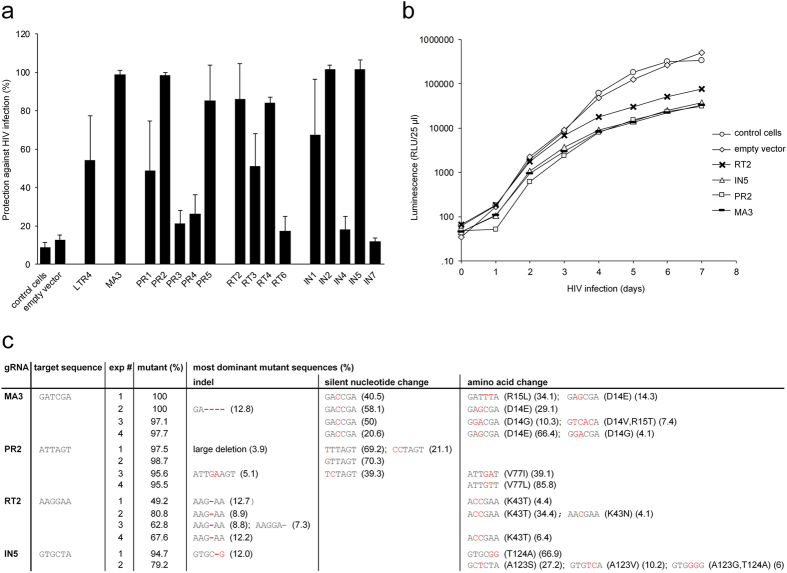
Single anti-HIV gRNAs cannot circumvent HIV replication and viral escape. (**a**) gRNAs targeting different regions of the HIV genome demonstrate protection against HIV infection in T cells. SupT1 cells stably transduced with a single gRNA were infected with HIV (MOI 0.006) and protection against HIV infection was determined after 4 days using an MTT based cell viability assay. Data is presented as a mean with standard deviation from three or more independent assays. (**b,c**) Single anti-HIV gRNAs cannot circumvent HIV replication and viral escape. SupT1 cells stably transduced with single gRNAs were infected with HIV-1 NanoLuc (MOI 0.006). Viral replication was monitored daily by analysis of luciferase activity. One representative experiment is shown from four independent assays (**b**). When full-blown infection of the SupT1 cells was observed, viral RNA was isolated from the supernatant, the target sites were amplified and subjected to deep-sequencing. Analysis of the gRNA target region (three nucleotides upstream and downstream of the cleavage site) is shown (**c**). Per individual experiment, the most frequently observed variants are indicated. Nucleotide substitutions and insertions and deletions in the target site sequence are presented in red.

Since we have shown that the CRISPR/Cas9 system is efficient in genome-editing and abrogation of viral expression we next investigated the potential to inhibit HIV replication in T cells. Specific gRNAs targeting HIV LTR (LTR4), matrix (MA3), protease (PR1-PR5), reverse transcriptase (RT2-RT4, RT6) and integrase (IN1, IN2, IN4, IN5, IN7) were generated and cloned in our lentiviral vector (Fig. 1a, Supplementary Table S1). SupT1 cells were transduced with the single gRNAs to assess their impact on HIV infection. After 4 days of HIV exposure, 97–100% protection against infection was observed for MA3, PR2, IN2 or IN5 (p < 0.0001) (Fig. 2a). gRNAs targeting the viral reverse transcriptase showed a maximum of 84% protection (RT2) against HIV infection (p = 0.001).

Subsequently, we investigated if these single gRNAs targeting different regions in the viral genome (MA3, PR2, RT2, IN5) could also prevent viral breakthrough. To this end, gRNA-expressing SupT1 cells were infected with HIV-1 reporter virus expressing a luciferase gene using an MOI of 0.006 and progression of the infection was followed in time by assessing reporter expression. Efficient viral replication was apparent in the control SupT1 cells and in SupT1 cells transduced with an empty vector (Fig. 2b). Remarkably, none of the single gRNAs could prevent breakthrough of viral replication (Fig. 2b), despite their initial potency to suppress HIV infection (Fig. 2a). In line with the reduced protection against HIV infection at early time points for the RT2 gRNA, we observed that virus infection progressed more efficiently for this gRNA as compared to gRNAs targeting MA3, PR2, and IN5 (Fig. 2b).

To investigate if the viruses replicating in the presence of the gRNAs were indeed viral breakthrough/escape variants, we amplified and deep-sequenced the CRISPR/Cas9 target region from multiple independent experiments. Interestingly, in the presence of gRNAs MA3, PR2 or IN5 a significantly higher percentage of the viral breakthrough population consisted of mutant sequences (79–100%) as compared to the sequences obtained from the viruses exposed to gRNA RT2 (49–81%) (p < 0.0001) (Fig. 2c, Supplementary Table S4). Selection of these mutant viruses in the presence of gRNAs MA3, PR2 or IN5 again underscores their strong inhibitory potential.

Analysis of the most dominantly observed mutant sequences revealed that MA3, PR2 or IN5 gRNAs predominantly resulted in the selection of sequences with nucleotide changes (silent and non-silent). For the less potent gRNA RT2, however, lower prevalence of mutant sequences were observed which predominantly consisted of variants with indels at the CRISPR/Cas9 target site (Fig. 2c, Supplementary Table S4). Taken together, this indicates that in the presence of the weak gRNA RT2 viral replication is still driven by the wildtype virus population whereas in the presence of the potent gRNAs viral replication is driven by escape mutants. The most dominant sequences obtained from the gRNA MA3 viral breakthrough population show either silent nucleotide changes or amino acid changes in the viral matrix protein (D14E/V/G and/or R15L/T). In case of gRNA PR2 the most dominant populations show mostly silent nucleotide changes or an amino acid change in the viral protease (V77I/L). No silent nucleotide changes were observed in the viral breakthrough population of gRNA IN5 or RT2, but amino acid changes were observed in HIV integrase (A123S/V/G and/or T124A) or reverse transcriptase (K43T/N), respectively.

Next, we investigated the frequency of off-target editing in the human genome for the same 3 gRNAs as used for the on-target analysis; LTR6, MA3 and IN5. For each gRNA, we selected the top three scoring off-target sites and designed primers to allow amplification of these sites from control SupT1 cells and SupT1 cells transduced with these gRNAs (Supplementary Table 2). Deep sequence analysis showed comparable levels of off-target genome editing events at the potential target sequence (defined as three nucleotides upstream and downstream of cleavage site) in the presence of gRNAs (0.43% to 1.73%) as in the control SupT1 cells that were not transduced with a gRNA (0.34% to 1.37%) (Supplementary Table 3). In addition, we investigated the impact of all single gRNAs on growth and viability of SupT1 cells. No impact of the stably expressed gRNAs was observed, except for gRNA LTR6 which severely reduced the viability of these cells and for that reason was excluded from further analysis (p < 0.0001; Supplementary Figure 1).

### Combinatorial CRISPR/Cas9 approach can prevent viral breakthrough

**Figure 3:**
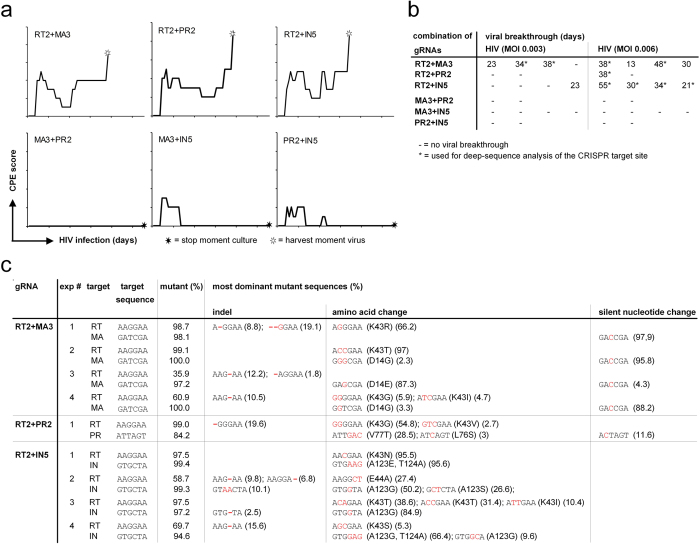
A combinational CRISPR/Cas9 gene-editing approach can halt HIV replication and prevent viral escape. SupT1 cells were stably transduced with combinations of two gRNAs (RT2-MA3; RT2-PR2, RT2-IN5, MA3-PR2, MA3-IN5 and PR2-IN5). These cells were infected with an HIV reporter virus using an MOI of 0.003 or 0.006. For two months, cultures were monitored daily for CPE and twice a week half of the culture was replaced by fresh culture media (**a,b**). A typical outcome of an experiment using a MOI of 0.006 was represented (**a**). When full-blown CPE was observed, viral RNA was isolated from the supernatant, the target sites were amplified and analyzed by deep-sequencing. Analysis of the gRNA target region (three nucleotides upstream and downstream of the cleavage site) is shown (**c**). Per individual experiment, the most frequently observed variants are indicated. Nucleotide substitutions and insertions and deletions in the target site sequence are presented in red.

Next, we investigated if a combination of two gRNAs targeting different steps in the viral life cycle could inhibit viral breakthrough in SupT1 cells. We transduced cells with two anti-HIV gRNAs and assessed virus replication upon HIV infection, using two different multiplicities of infection, by monitoring cytopathogenic effect (CPE) in time (Fig. 3a). We monitored CPE instead of the potentially more sensitive analysis of luciferase, because we noticed that luciferase could still be expressed from defective proviral DNA, even if we added T20 as a control.

Interestingly, irrespective of the MOI used, combinations consisting of two strong inhibitory gRNAs (MA3 + PR2, MA3 + IN5 and PR2 + IN5) efficiently abrogated virus replication, whereas combinations of a weak (RT2) and a strong gRNA could not (Fig. 3a). We did observe minor effects on cytopathogenicity for the strong inhibitory gRNA combinations at early time points, whereas after prolonged culture virus-induced CPE disappeared completely. These cultures remained HIV negative for two months (Fig. 3a), independent of initial viral dosage (Fig. 3b). In contrast, non-optimal gRNA combinations resulted in a viral breakthrough in 4 out of 10 experiments using a low HIV infectious dose (MOI 0.003) and 9 out of 10 experiments using a higher infectious dose (MOI 0.006) (Fig. 3a,b). Analysis of proviral DNA encompassing the neighboring RT2 and PR2 target sites showed that about 15% of the proviral DNA contains an exact deletion between the two target sites and even more sequences contain a larger deletion. Despite this deleterious effect of the CRISP/Cas9 endonuclease, non-optimal combinations of gRNAs only partly control virus infection, ultimately resulting in viral breakthrough.

To investigate if the viruses replicating in the presence of these non-optimal gRNA combinations were indeed viral breakthrough/escape variants, we next amplified and deep-sequenced their CRISPR/Cas9 target regions. In the viral breakthrough population, a high percentage of mutant sequences were observed in the target regions of the strong gRNAs MA3 (97–100%), IN5 (94–99%) and PR2 (84%) (Fig. 3c; Supplementary Table S4). The dominant sequences in these populations did not contain indels but rather contained silent nucleotide changes or amino acid changes at the target sites (Matrix: D14G/E; Protease: V77T or L76S; Integrase: A123E/G/S and/or T124A). In the background of these mutant viral populations in the MA3, IN5 and PR2 target sites varying percentages of mutant sequences were observed (36–99%) in the gRNA RT2 target region. These high percentages of mutant sequences at both target sites imply that most of these gRNA escape mutations are present on one viral genome. More directly, in the case of the PR2-RT2 experiment we amplified a region encompassing both gRNA target sites and demonstrated that both gRNA escape mutations were indeed present in the same viral genomic sequence. In all experiments where high percentages of RT2 mutant sequences were detected, they most dominantly represented amino acid changes (K43R/T/G/I/V/N/S or E44A) and not indels. In contrast, in the experiment where just 36% mutant RT sequences were present, only indels in the RT2 target region were observed in the dominant populations.

## Discussion

This study demonstrates that a combinatorial CRISPR/Cas9 gene-editing approach, targeting two steps in the viral lifecycle, can halt viral replication and prevent escape.

Gene editing based on the CRISPR/Cas9 system can very efficiently target, inactivate and even delete HIV DNA from latently infected cells and limit the infection of new cells^35^,^36^,^37^,^38^,^39^,^40^,^41^. However, very little is known about the long-term inhibitory effects of this approach and especially whether and how resistance may develop. During a CRISPR/Cas9 attack, the HIV DNA sequence is targeted by the gRNA and the proviral DNA is cleaved by the Cas9 endonuclease. Subsequently, the cellular error-prone non-homologous end joining (NHEJ) machinery will try to repair the DNA break and generate mutations at the cleavage site^16^. Insertions and deletions are hallmark of NHEJ action, but also nucleotide substitutions are generated. Two very recent publications have shown that the NHEJ repair machine may facilitate HIV escape^42^,^43^. They demonstrate that HIV can rapidly and consistently escape the inhibitory effect of a single gRNA based CRISPR/Cas9 attack. Sequencing of the viral escape variants revealed nucleotide insertions, deletions and substitutions around the Cas9 cleavage site that are typical for DNA repair by the NHEJ machinery. These observations questioned the feasibility of the CRISPR/Cas9 system based gene-editing technology as an approach to combat HIV infections^44^,^45^.

For this study, we have combined our knowledge on the CRISPR/Cas9 system to edit viral genomes^25^ with our experience in HIV antiretroviral drug escape^46^,^47^,^48^ to investigate if HIV can overcome a (combinatorial) CRISPR/Cas9 approach. We generated several gRNAs targeting different regions of the viral genome and steps in the viral life cycle. In latently infected cells, we observed very efficient targeting and editing of the viral DNA, resulting in abrogation of HIV gene expression. We also demonstrated that these single gRNAs could suppress HIV infection, but were unable to prevent a viral breakthrough in T-cells. In general, during *in vitro* selection experiments using antiretroviral compounds or RNAi-based inhibitory approaches, a G-A nucleotide substitution is the most commonly observed escape mutation in the viral population^49^,^50^. This substitution is likely generated by the error-prone HIV-RT and cellular factors such as APOBEC3G^51^,^52^. In line with two recent publications on single gRNA induced HIV escape^42^,^43^, we observed not only G-A substitutions (5 G-A) but mostly other single nucleotide mutations (37 A-G; 31 T-C; 24 T-G; 5 G-A) (Supplementary Table S5). This indicates that on top of the errors introduced by HIV and potential cellular factors, the CRISPR/Cas9 endonuclease functions as a DNA sequence-specific mutagen accelerating the generation of viral mutants in the target region thereby enhancing viral breakthrough. This fast escape is remarkable, since the generation of sequence variation and subsequent outgrowth of mutants with improved fitness, usually takes several weeks or months for antiretroviral compounds tested in our experimental system. The fact that most viruses escape with just a single (silent) nucleotide substitution in the target site of the CRISPR/Cas9 endonuclease underlines the sequence specificity of the endonuclease. This sequence specificity will reduce the chances of off-targeting of this editing strategy.

Simultaneous cleavage at two target sites within the HIV provirus can result in large deletions of the intervening region of the HIV genome, thereby completely disrupting HIV replication, as we have observed in this study and was previously reported when targeting an LTR region that is present at both ends of the HIV proviral genome^35^,^36^,^39^,^40^,^41^. In our analysis we PCR amplified the relevant gRNA target regions prior deep-sequencing, thereby potentially missing information on the prevalence of these large deletions. However, as a large deletion in the proviral genome renders these variants completely inactive, they will not provide an opportunity for viral escape. Inefficient cleavage of one of the two gRNAs, however, will not result in the formation of these large deletions, but rather result in editing of one of the two target sites. Replication of newly emerged virus variants may be targeted at the other gRNA target site in the subsequent rounds of infection. Our analysis focusses on these escape mutants.

Differences in potency of the single gRNAs may be the related to the level of conservation of the viral genome and/or the result of variances in the efficiency of Cas9 cleavage at the individual target sites. The potency of some of our single gRNAs (MA3, PR2, IN5) is reflected by the observation that the viral escape population is almost completely dominated by mutant viruses and hardly any wildtype HIV is observed. Likewise, in the early days of antiretroviral therapy using single RT-inhibitors, the strong inhibitory potential of lamivudine (3TC) was reflected by the very fast appearance of mutant viruses that dominated the viral population^53^,^54^.

As well as with HIV therapy, the combinatorial use of multiple gRNAs proved essential to prevent the generation and selection of resistance. We showed that a combination of two potent gRNAs completely abrogated viral replication, which could not be rescued during several months of continuous selection. For this study, we have only investigated combinations of gRNAs targeting different regions of the viral genome and different steps in the viral lifecycle. It would be interesting to investigate if a combination of two potent inhibitors targeting the same step in the viral lifecycle, would provide the same protection against viral breakthrough. It would also be interesting to investigate if this editing strategy would prevent viral escape in *ex vivo* and/or *in vivo* models. We have shown that non-optimal combinations of gRNAs, consisting of a potent and a less-potent gRNA can only partly control HIV infection, ultimately resulting in a viral breakthrough. Like with cART, where the cART regimen is adapted based on a high baseline viral load, gRNA combinations may have to be customized based on the size and genetic constitution of the viral population that needs to be controlled. Taken together, our data shows that the accelerating effect of CRISPR/Cas9 genome-engineering on viral escape can be overcome and as such gene-editing may provide a future alternative for control of HIV-infection.

## Experimental Procedures

### Cell culture

Human embryonic kidney (HEK) cell line 293 T^55^, human T lymphoblast cell line SupT1^56^, J.Lat Full Length Clone 15.4 (J.Lat FL cells; Jurkat cells containing near full length HIV-1 (without env and nef)) were obtained from the NIH AIDS Reagent Program, Division of AIDS, NIAID. HEK-293T cells were maintained in DMEM (Lonza), supplemented with 10% fetal bovine serum (FBS)(Sigma-Aldrich) and 10 μg/ml gentamicin (Invitrogen). All other cells were cultured in RPMI1640 with L-glutamine (Lonza), 10% FBS and 10 μg/ml gentamicin.

### CRISPR-Cas9 vector construct

For efficient transduction and stable expression of the CRISPR/Cas9 system in human cell lines, we developed a selectable lentiviral CRISPR/Cas vector^57^ to facilitate efficient and selectable expression of Cas9 and gRNAs in target cells: briefly, the 3rd generation lentiviral pSicoR vector (Jacks Lab, MIT) was altered to express a nuclear-localized Cas9 gene that was N-terminally fused to either BlastR or PuroR and a T2A sequence. Additionally, the region immediately downstream of the U6 promoter was replaced by a cassette consisting of two unique restriction sites (BsmBI) to allow cloning of crRNA target sites fused to a tracrRNA sequence and a terminator consisting of 5 T-residues. The vectors were called pSicoR-CRISPR-PuroR and pSicoR-CRISPR-BlastR.

Target sequences were designed based on a reference HIV genome sequence (HXB2, HIV sequence database, Los Alamos) and all sequences (20 nts) initiated with a G-residue to allow for potent expression from the U6 promoter. Since the HIV genome has a low GC-content, we allowed for the presence of both NGG and NAG PAM sequences adjacent the target site to increase the likelihood of identifying potent anti-HIV gRNAs (Fig. 1a, Supplementary Table S1). Potent anti-HIV gRNAs were analyzed by the “Optimized CRISPR Design” tool from the Zhang lab (crispr.mit.edu) to identify potential off-target sites in the human genome. Upon cloning of crRNA sequences in the pSicoR-CRISPR vectors, single bacteria colonies were picked, expanded and isolated using the GeneJET Plasmid Miniprep Kit (Thermo Scientific). The vector constructs were sequence-verified using primer EF1a_rev: 3′TCTAGGCACCGGGTCAATTGC5′ and Big-dye terminator sequencing kit 1.1 (Life Technologies). For this study we continued with those constructs in which the gRNA sequences were correctly introduced (LTR4, LTR6, MA3, PR1-PR5, RT2-RT4, RT6, IN1, IN2, IN4, IN5 and IN7; Supplementary Table S1). Of note, we observed that our lentiviral pSicoR-CRISPR plasmids containing anti-LTR gRNAs did not give rise to clean single-sequence plasmid preps. As the pSicoR-CRISPR vector backbone holds HIV LTR sequences that are compatible with the anti-LTR gRNAs cloned downstream of the U6 promoter, we speculate that some gRNA expression may occur in bacteria that result in targeting of the plasmid backbone during the culture. The lower quality DNA preps were only observed for anti-LTR sequences, and not for any of the other anti-HIV gRNAs.

### Lentivirus production

Lentiviruses were packaged using Mirus (Mirus Bio) transfection of HEK-293T cells with the abovementioned vector in combination with standard lentiviral packaging vectors and harvested 72 hours post transfection.

### HIV-1 reporter virus

To generate a reporter virus (HIV-1 NanoLuc), the NanoLuc luciferase gene (pNL1.3)(Promega) was cloned into the nef gene of our HIV-1 reference clone HXB2 using the unique restriction site Bpu1102I. In brief, two gBlock gene fragments (Integrated DNA Technologies, IDT) encompassing the NanoLuc gene, including a start and stop codon, and harboring the Bpu1102I restriction site on both ends were assembled (Gibson Assembly kit, new England Biolabs) and amplified using the Expand High Fidelity PCR system (Roche) and the Lucrevbpu1102I 5′-CTGCTGCTGGCTCAGCCT-3′ and Lucforbpu1102I 5′-TGAGACGAGCTGAGCATG-3′ primers, identical to the 5′ and 3′ ends of the assembled fragment. The amplified product was cloned in the HXB2 molecular clone using the unique Bpu1102I restriction site. Single colonies were picked, expanded, plasmid DNA was isolated using the GeneJET Plasmid Miniprep Kit (Thermo Scientific) and sequenced using primers 5′Nef-1 5′-GCAGTAGCTGAGGGGACAGA-3′ and lucseq1 5′-CAGTCTTCACACTCGAAG-3′ and the Big-dye terminator sequencing kit 1.3 (Life Technologies).

To obtain the reporter virus HIV-1 NanoLuc, 10 μg of the HXB2 luciferase plasmid was used to transfect HEK-293T cells at 90–95% confluence using lipofectamine 2000 reagent (Invitrogen). After 48 h, supernatant was harvested and used for analysis of luciferase activity and 50% tissue culture infective dose (TCID_50_) on SupT1 cells.

### HIV Reactivation in T cell latency model

J.Lat Full Length Clone 15.4 (Jurkat cells containing near full length HIV-1 expressing GFP) serves as a common HIV-1 latency model for T cells^58^. We transduced 40,000 of these target cells using 50–250 μl lentiviral supernatants via spinoculation at 1,000 g for 90 minutes at 33 °C. After 2–3 days, we selected for successfully transduced cells using either 2 μg/ml puromycin (Sigma-Aldrich) or 20 μg/ml blasticidin (Bio-connect). Double gRNA expressing cell lines were generated by initial transduction using the appropriate pSicoR-CRISPR-PuroR vector, followed by puromycin selection and subsequent transduction with the appropriate pSicoR-CRISPR-BlastR vector and blasticidin selection. Next, HIV was reactivated from 40,000 transduced cells using 0.1, 1 and 5 ng/ml TNF-α (Life Technologies). After 48 hours, GFP expression was analyzed by flow cytometry.

### gRNA on-target mutational analysis in latency model

To study the frequency of target site mutations due to CRISPR/Cas9 editing, Jurkat cells containing a near full length copy of HIV (J.Lat Full Length Clone 15.4; HIV-R7/E-/GFP)^58^ were transduced with lentiviruses containing a CRISPR/Cas9 gRNA targeting the LTR region (LTR6), the structural protein matrix (MA3) or the integrase enzyme (IN5). 40,000 cells were transduced with using 50–250 μl lentiviral supernatants via spinoculation at 1,000 g for 90 minutes at 33 °C. After 2–3 days, successfully transduced cells were selected for by using 2 μg/ml puromycin (Sigma-Aldrich). Genomic DNA from 5.10^6^ lentivirus infected cells and uninfected control cells was isolated using the DNeasy Blood & Tissue Kit (Qiagen). Samples were eluted in RNAse free H_2_O and stored at −20 °C. Target sites were amplified using Platinum^®^ Taq DNA Polymerase High Fidelity (Life Technologies) and primers with specific 454 linker and barcode sequences (Supplementary Table S2). The PCR product was subsequently purified using the GeneJET PCR Purification Kit (Thermo Scientific).

### gRNA off-target mutational analysis

To study the frequency of off-target editing in the human genome, we assessed the potential occurrence of off-target mutations for the same 3 gRNAs as used for the on-target analysis; LTR6, MA3 and IN5. For each gRNA, we selected the top three scoring potential off-target sites as assessed by the Zhang algorithm (crispr.mit.edu) and designed primers using the primer3web (http://bioinfo.ut.ee/primer3/) to allow amplification of these sites (Supplementary Table S2). Genomic DNA from 5.10^6^ lentivirus infected SupT1 cells and uninfected SupT1 cells was isolated using the DNeasy Blood & Tissue Kit (Qiagen). Samples were eluted in RNAse free H_2_O and stored at −20 °C. Target sites were amplified using Platinum^®^ Taq DNA Polymerase High Fidelity (Life Technologies) and primers with specific 454 linker and barcode sequences. The PCR product was subsequently purified using the GeneJET PCR Purification Kit (Thermo Scientific).

### Analysis of prevention of HIV infection in SupT1 cells

We transduced 40,000 SupT1 cells using 50–250 μl lentiviral supernatants via spinoculation at 1,000 g for 90 minutes at 33 °C. After 2–3 days, we selected for successfully transduced cells using either 2 μg/ml puromycin (Sigma-Aldrich) or 20 μg/ml blasticidin (Bio-connect). We infected 40,000 lentivirus transduced SupT1 cells in triplicate with the HIV-1 NanoLuc reporter virus using an MOI of 0.006. Cells and virus were cultured for 4 days at 37 °C. At day 4, protection against HIV infection was measured using a cell viability assay.

### Analysis of viral breakthrough in SupT1 cells expressing a single gRNA

We transduced 40,000 SupT1 cells using 50–250 μl lentiviral supernatants via spinoculation at 1,000 g for 90 minutes at 33 °C. After 2–3 days, successfully transduced cells were selected with either 2 μg/ml puromycin (Sigma-Aldrich) or 20 μg/ml blasticidin (Bio-connect). Next, transduced SupT1 cells were infected for 2 hours at 37 °C with HIV-1 NanoLuc reporter virus using an MOI of 0.006. After infection, the cells were washed twice and 60 μl of culture supernatant was isolated and stored at −20 °C on a daily basis for subsequent luciferase activity assays. When full-blown CPE was observed, cell free virus was harvested. After DNase treatment of the cell free culture supernatant, viral RNA was isolated and used to investigate viral escape at the gRNA target site. In brief, 200 μl of culture supernatant was treated with 2 μl TURBO DNase (Life Technologies) for 90 minutes in Turbo buffer at 37 °C, after which viral RNA was isolated^59^. Viral RNA was converted into cDNA and amplified using a one-step RT-PCR reaction (SuperScript^®^ III One-Step RT-PCR System with Platinum^®^ Taq DNA Polymerase) (Invitrogen) and on-target primers with specific 454 linker and barcode sequences. The PCR product was subsequently purified using the GeneJET PCR Purification Kit (Thermo Scientific).

### Analysis of viral breakthrough in SupT1 cells expressing two gRNAs

We transduced 40,000 SupT1 cells using 50–250 μl lentiviral supernatants via spinoculation at 1,000 g for 90 minutes at 33 °C. After 2–3 days, we selected for successfully transduced cells using either 2 μg/ml puromycin (Sigma-Aldrich) or 20 μg/ml blasticidin (Bio-connect). Double gRNA expressing cell lines were generated by initial transduction using the appropriate pSicoR-CRISPR-PuroR vector, followed by puromycin selection and subsequent transduction with the appropriate pSicoR-CRISPR-BlastR vector and blasticidin selection. Next, these transduced SupT1 cells were infected for 2 hours at 37 °C with our reporter virus HIV-1 NanoLuc using an MOI of 0.003 or 0.006. For two months, cultures were monitored daily for CPE and twice a week half of the culture was replaced by fresh culture media. We monitored CPE instead of the potentially more sensitive analysis of luciferase, because we noticed that luciferase could still be expressed from defective proviral DNA, even if we added T20 (1 μM) as a control. As such analysis of luciferase was not a good measurement of ongoing (low level) viral replication and escape. When full-blown CPE was observed, cell free virus was harvested. Viral escape at the gRNA target sites was analyzed as described above (Analysis of viral breakthrough in SupT1 cells expressing a single gRNA).

### Deep-sequencing analysis

The DNA concentration of the purified amplicons of the on-target and off-target PCR analysis was determined using the Quant-iT PicoGreen dsDNA Assay kit (Invitrogen). To allow discrimination between amplicons from control and CRISPR-expressing cells, we designed primer-sets for the different target areas by adding a 454 linker and a unique barcode to the 5′ end of the primers. All PCR products were diluted to a concentration of 10^9^ molecules/μl and amplicons from gRNA-expressing cells and control cells were mixed in a 2:1 ratio mixed to form the sample library and sequenced using the GS Junior Titanium emPCR Kit (Lib-A) (Roche) and the GS Junior sequencer (Roche). Sequences were analyzed using GS Amplicon Variant Analyzer software (Roche) and MEGA 6^60^. The CRISPR/Cas9 target region was defined as three nucleotides upstream and downstream of cleavage site.

### Analysis of luciferase activity

Luciferase activity of HIV-1 NanoLuc was assessed according to the manufacturer’s protocol using the Nano-Glo^®^ Luciferase Assay System (Promega). In brief, 25 μl of culture supernatant was mixed with the same volume of Nano-Glo assay mix in a white 96 well half area microplate (Greiner Bio-One) and luminescence was measured after 5 minutes using a TriStar LB 941 Multimode Microplate Reader (Berthold Technologies).

### GFP flow cytometry

Cells were washed with PBS and fixed for 10 minutes at room temperature in PBS supplemented with 1% formaldehyde. Cells were subsequently analyzed using a FACSCANTO II flow cytometer (BD Biosciences). Data was analyzed using FloJo (v7.6.3).

### Cell viability analysis

Cell viability was measured in an MTT (3-(4,5-Dimethylthiazol-2-yl)-2,5-Diphenyltetrazolium Bromide) (Sigma) assay. Actively respiring cells convert the water-soluble MTT to an insoluble purple formazan which can be solubilized with 0.4% HCL, 10% Triton in isopropanol and its concentration can be measured at a wavelength of 540 nm (690 nm as reference wavelength).

### Statistical analysis

Data is presented as a mean with standard deviation from three or more independent assays. All statistical comparisons were evaluated by an unpaired t-test.

## Additional Information

**How to cite this article**: Lebbink, R. J. *et al*. A combinational CRISPR/Cas9 gene-editing approach can halt HIV replication and prevent viral escape. *Sci. Rep.* **7**, 41968; doi: 10.1038/srep41968 (2017).

**Publisher's note:** Springer Nature remains neutral with regard to jurisdictional claims in published maps and institutional affiliations.

## Supplementary Material

Supplementary Information
