# Supplementary material for: A combinational CRISPR/Cas9 gene-editing approach can halt HIV replication and prevent viral escape
Source: Sci Rep. 2017 Feb 8;7:41968. doi: 10.1038/srep41968 (PMC5296774; doi:10.1038/srep41968)

Supplementary information

A combinational CRISPR/Cas9 gene-editing approach can halt HIV replication and prevent viral escape

Robert Jan Lebbink, Dorien CM de Jong, Femke Wolters, Elisabeth M Kruse, Petra M van Ham, Emmanuel JHJ Wiertz and Monique Nijhuis*

Department of Medical Microbiology, Virology, University Medical Center Utrecht, Utrecht, The Netherlands

Supplementary Table S1. gRNA sequences used in this study.

Supplementary Table S2. Primer sequences for on-and off-target analysis.

Supplementary Table S3. Analysis of off-target activity of anti-HIV gRNAs.

Supplementary Table S4. Deep sequence analysis of gRNA target sites after (long-term) in vitro selection experiments.

Supplementary Table S5. Nucleotide substitution patterns observed in HIV escape variants.

Supplementary Figure S1. Impact of Cas9/gRNA expression on cell growth and viability. SupT1 cells were stably transduced with the single gRNAs. After four days, cell growth and viability was measured in an MTT based cell viability assay. Actively respiring cells convert the water-soluble MTT to an insoluble purple formazan which can be solubilized and its concentration can be measured at a wavelength of 540 nm. Data is presented as a mean with standard deviation from three or more independent assays.

Table S1. Selected gRNA sequences used in this study

CRISPR ID	target gene	gRNA sequence	PAM
**GFP1**	green fluorescent protein	TGAACCGCATCGAGCTGAAG	GGG
**LTR4**	HIV-LTR	GCCACTCCCCAGTCCCGCCC	AGG
**LTR6**	HIV-LTR	GCTCAGATCTGGTCTAACCA	GAG
**MA3**	HIV matrix (p17)	GCGGGGGAGAATTAGATCGA	TGG
**PR1**	HIV protease	GATGGAAACCAAAAATGATA	GGG
**PR2**	HIV protease	GCTATAGGTACAGTATTAGT	AGG
**PR3**	HIV protease	GTATCTAATAGAGCTTCCTT	TAG
**PR4**	HIV protease	GCAACTAAAGGAAGCTCTAT	TAG
**PR5**	HIV protease	GGAAGCTCTATTAGATACAG	GAG
**RT2**	HIV reverse transcriptase	GTACAGAGATGGAAAAGGAA	GGG
**RT3**	HIV reverse transcriptase	GGAGTATTGTATGGATTTTC	AGG
**RT4**	HIV reverse transcriptase	GACTTCTGGGAAGTTCAATT	AGG
**RT6**	HIV reverse transcriptase	GTCTGGATTTTGTTTTCTAA	AAG
**IN1**	HIV integrase	GATATTTCTCATGTTCATCT	TGG
**IN2**	HIV integrase	GGACAAGTAGACTGTAGTCC	AGG
**IN4**	HIV integrase	GTTTCCTGCCCTGTTTCTGC	TGG
**IN5**	HIV integrase	GGCGGCCTTAACCGTAGCAC	TGG
**IN7**	HIV integrase	GCCGGTAAAAACAATACATA	CAG

Table S2. Primer sequences for on- and off-target analysis

	On - off target sequence	mismatches + location	Chr.	position	Fw primer (5'-3')	Rev primer (5'-3')
**LTR6 ON**	GCTCAGATCTGGTCTAACCA**GAG**				AGAACTGCTGAcATCGAGCTTGC	CTTCAGCAAGCCGAGTCCTG
**LTR6 OFF1**	GCCCAGAGCCGGTCTAACCA**GAG**	3MMs [3:8:10]	20	-19351498	TGCCAACCTATCCCAGCAGG	AGACCCACACCAAGACAGGC
**LTR6 OFF2**	TCCAAGAACTGGTCTAACCA**AGG**	4MMs [1:3:4:8]	4	3277031	ACCCTTTCACAACCACCAGC	AGCCCTGACTTCATTGTGCAAC
**LTR6 OFF3**	GTGCAGACCTTGTCTAACCA**AGG**	4MMs [2:3:8:11]	9	-112431084	TTTGGTCTGTGGGCAAACTG	CCAGTGCTAAGCAGACTTGAGG
**MA3 ON**	GCGGGGGAGAATTAGATCGA**TGG**				CTCTCTCGACGCAGGACTCG	TGTGTCCTGTGTCAGCTGCT
**MA3 OFF1**	TCCAGGGAGAATCAGATCGA**TGG**	4MMs [1:3:4:13]	3	-193038771	TCTTATACATCTGGCTGCCGC	ACCACAGTGAGGCATCTGGG
**MA3 OFF2**	CCTGTGGAGAATTAGATCTA**AGG**	4MMs [1:3:5:19]	4	76175203	CTGGCACACTGACAAAGCAG	GAAAAAGGCACCCTGTCTGG
**MA3 OFF3**	GAGGGGGAGAATGAGAACGA**TGG**	3MMs [2:13:17]	X	40589720	GCCAGCAGACCGGAGAGATG	TAGGGGAAAGGGGAGGCAGG
**IN5 ON**	GGCGGCCTTAACCGTAGCAC**TGG**				GCCAGTGGATATATAGAAGC	CCAATCCCCCCTTTTCTTT
**IN5 OFF1**	GGAGGACGTAACTGTAGCAC**TAG**	4MMs [3:6:8:13]	18	-75412696	CTGCTCAGACTGTGCCATCC	TTCCTCAGAGGTTATTCTTTCAATG
**IN5 OFF2**	GGCAGCCTGCACTGTAGCAC**TAG**	4MMs [4:9:10:13]	10	119015953	CCCAGTGGTAGGAGGCTGC	AGGGTCTTGGCCAGGGTTAC
**IN5 OFF3**	TACTGCCTTAACCGTGGCAC**AAG**	4MMs [1:2:4:16]	7	93632950	GCCGTTCTAGTAGCTGTGCG	TGTTTCCCTACTGGCCCTGG

Table S3. Analysis of off-target activity of anti-HIV gRNAs

		gRNA positive cells			gRNA negative cells			
		Sequences			Sequences			
gRNA		Total	Mutant (%)	Wildtype (%)	Total	Mutant (%)	Wildtype (%)	gRNA pos – gRNA neg (%)
**LTR6**	Off1	2491	0.60	99.40	1241	1.37	98.63	-0.77
	Off2	4177	1.01	98.99	1166	0.86	99.14	0.15
	Off3	2426	0.91	99.09	951	1.05	98.95	-0.14
**MA3**	Off1	2259	1.73	98.27	1111	0.81	99.19	0.92
	Off2	2007	0.90	99.10	977	0.34	99.66	0.56
	Off3	1841	0.43	99.57	770	1.17	98.83	-0.74
**IN5**	Off1	1743	1.09	98.91	1019	0.59	99.41	0.50
	Off2	2487	0.88	99.12	1087	0.65	99.35	0.23
	Off3	2903	0.76	99.24	1374	0.66	99.34	0.10

Table S4. Deep sequence analysis of gRNA target sites after (long-term) in vitro selection experiments

		PR			RT			IN			MA		
**gRNA**	**exp #**	**total count**	**seq**	**%**	**total count**	**seq**	**%**	**total count**	**seq**	**%**	**total count**	**seq**	**%**
**control cells**	2	864	**ATTAGT**	99,8	864	**AAGGAA**	98,8	**904**	**GTGCTA**	100,0	235	**GATCGA**	**100,0**
**empty vector**	2	748	**ATTAGT**	99,7	748	**AAGGAA**	99,7	**873**	**GTGCTA**	100,0	239	**GATCGA**	**100,0**
**PR2**	1	565	**ATTAGT**	**2,5**									
			TTTAGT	69,2									
			CCTAGT	21,1									
			22 del	3,9									
**PR2**	2	829	**ATTAGT**	**1,3**									
			GTTAGT	70,3									
**PR2**	3	806	**ATTAGT**	**4,4**									
			TCTAGT	39,3									
			ATTGAT	39,1									
			ATTGAAGT	5,1									
**PR2**	4	776	**ATTAGT**	**4,5**									
			ATTGTT	85,8									
**RT2**	1				1532	**AAGGAA**	**50,8**						
						AAG-AA	12,7						
						ACCGAA	4,4						
**RT2**	2				914	**AAGGAA**	**19,2**						
						ACCGAA	34,4						
						AAG-AA	8,9						
						AACGAA	4,1						
**RT2**	3				1063	**AAGGAA**	**37,2**						
						AAG-AA	8,8						
						AAGGA-	7,3						
**RT2**	4				1268	**AAGGAA**	**32,4**						
						AAG-AA	12,2						
						ACCGAA	6,4						
**IN5**	1							837	**GTGCTA**	**5,3**			
									GTGCGG	66,9			
									GTGC-G	12,0			
**IN5**	2							572	**GTGCTA**	**20,8**			
									GCTCTA	27,2			
									GTGTCA	10,2			
									GTGGGG	6,0			
**MA3**	1										127	**GATCGA**	**0,0**
												GACCGA	40,5
												GATTTA	34,1
												GAGCGA	14,3

**MA3**	2										149	**GATCGA**	**0,0**
												GACCGA	58,1
												GAGCGA	29,1
												GA----	12,8
**MA3**	3										311	**GATCGA**	**2,9**
												GACCGA	50,0
												GGACGA	10,3
												GTCACA	7,4
**MA3**	4										442	**GATCGA**	**2,3**
												GAGCGA	66,4
												GACCGA	20,6
												GGACGA	4,1
**RT2 + PR2**	1	932	**ATTAGT**	**15,7**	932	**AAGGAA**	**1,0**						
			ATTGAC	28,5		GGGGAA	54,8						
			ACTAGT	11,6		-GGGAA	19,6						
			ATCAGT	3,0		GTCGAA	2,7						
**RT2 + IN5**	1				775	**AAGGAA**	**2,5**	894	**GTGCTA**	**0,6**			
						AACGAA	95,5		GTGAAG	95,6			
**RT2 + IN5**	2				502	**AAGGAA**	**41,3**	686	**GTGCTA**	**0,7**			
						AAGGCT	27,4		GTGGTA	50,2			
						AAG-AA	9,8		GCTCTA	26,6			
						AAGGA-	6,8		GTAACTA	10,1			
**RT2 + IN5**	3				530	**AAGGAA**	**2,5**	691	**GTGCTA**	**2,8**			
						ACAGAA	38,6		GTGGTA	84,9			
						ACCGAA	31,4		GTG-TA	2,5			
						ATTGAA	10,4						
**RT2 + IN5**	4				629	**AAGGAA**	**30,3**	501	**GTGCTA**	**5,4**			
						AAG-AA	15,6		GTGGAG	66,4			
						AGCGAA	5,3		GTGGCA	9,6			
**RT2 + MA3**	1				777	**AAGGAA**	**1,3**				634	**GATCGA**	**1,9**
						AGGGAA	66,2					GACCGA	97,9
						--GGAA	19,1						
						A-GGAA	8,8						
**RT2 + MA3**	2				1296	**AAGGAA**	**0,9**				432	**GATCGA**	**0,0**
						ACCGAA	97,0					GACCGA	95,8
												GGGCGA	2,3
**RT2 + MA3**	3				1441	**AAGGAA**	**64,1**				645	**GATCGA**	**2,8**
						AAG-AA	12,2					GAGCGA	87,3
						-AGGAA	1,8					GACCGA	4,3
**RT2 + MA3**	4				506	**AAGGAA**	**39,1**				363	**GATCGA**	**0,0**
						AAG-AA	10,5					GACCGA	88,2
						GGGGAA	5,9					GGTCGA	3,3
						ATCGAA	4,7						

Table S5. Nucleotide substitution patterns in HIV escape variants

HIV target gene	PR	RT	IN	MA	Total
Samples (n)	14	21	14	18	67
target sequence	ATTAGT	AAGGAA	GTGCTA	GATCGA	
A-->T	4	5	0	1	10
A-->C	2	13	0	0	15
A-->G	10	10	7	10	37
T-->A	0	0*	5	5	10
T-->C	13	0*	5	13	31
T-->G	1	0*	5	18	24
C-->A	0*	0*	1	1	2
C-->T	0*	0*	1	1	2
C-->G	0*	0*	11	0	11
G-->A	2	1	1	1	5
G-->T	1	2	3	1	7
G-->C	1	15	1	1	18
	34	46	40	52	172

* Nucleotide is not present in target site

Figure S1. Impact of Cas9/gRNA expression on cell growth and viability

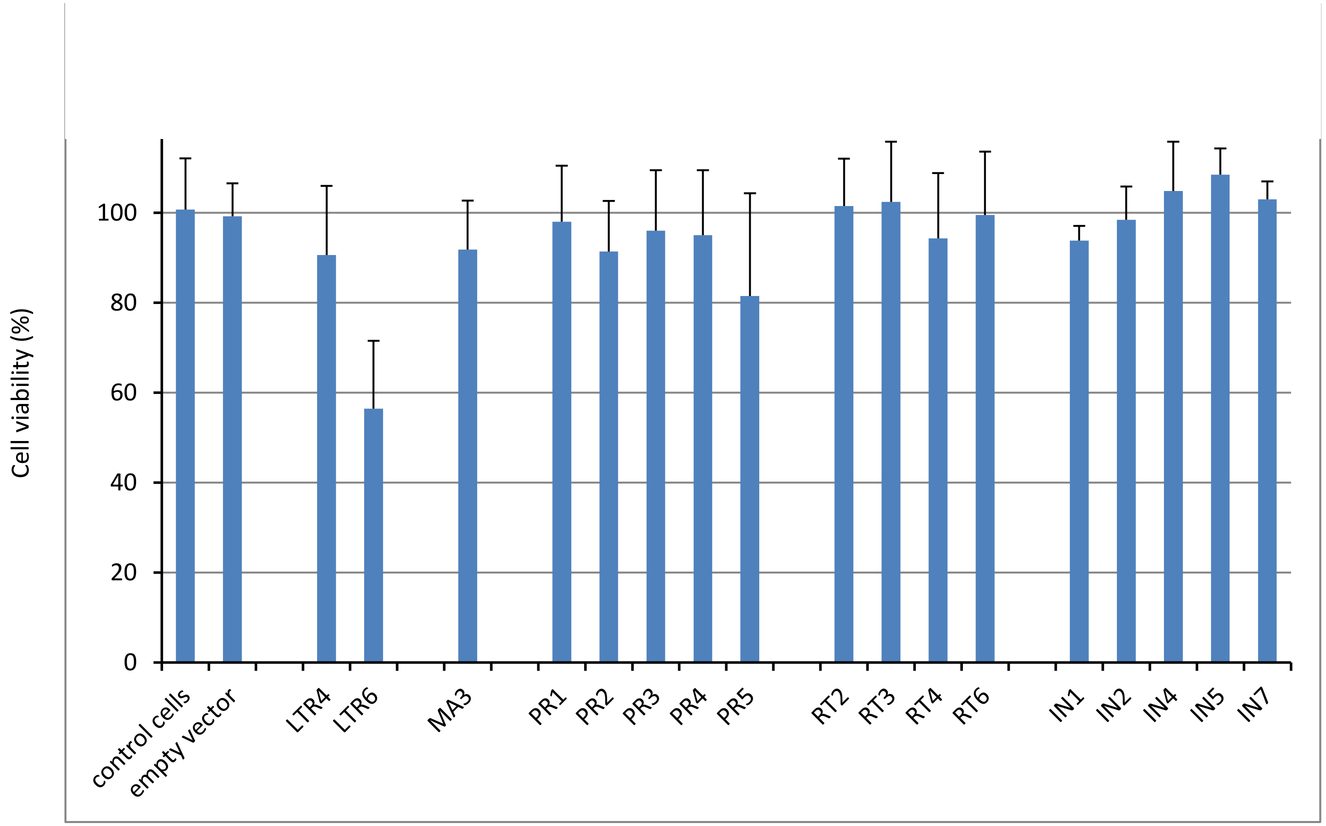

Supplement: Supplementary Information [file srep41968-s1.pdf]
